# The Highly Efficient Synthesis of 1,2-Disubstituted Benzimidazoles Using Microwave Irradiation

**DOI:** 10.3390/molecules27051751

**Published:** 2022-03-07

**Authors:** Monica Nardi, Sonia Bonacci, Natividad Herrera Cano, Manuela Oliverio, Antonio Procopio

**Affiliations:** 1 Dipartimento di Scienze della Salute, Università Magna Græcia, Viale Europa, Germaneto, 88100 Catanzaro, CZ, Italy; s.bonacci@unicz.it (S.B.); m.oliverio@unicz.it (M.O.); procopio@unicz.it (A.P.); 2 ICYTAC, Instituto de Ciencia y Tecnología de Alimentos de Córdoba, CONICET, Departamento de Química Orgánica, Facultad de Ciencias Químicas, Universidad Nacional de Córdoba, Ciudad Universitaria, Bv. Juan Filloy s/n, Córdoba 5000, Argentina; nhc@fcq.unc.edu.ar

**Keywords:** microwave, green chemistry, benzimidazoles, solvent free conditions, Er(OTf)_3_

## Abstract

The benzimidazole ring of the heterocyclic pharmacophores is one of the most widespread and studied systems in nature. The benzimidazole derivative synthesis study is a crucial point for the development of a clinically available benzimidazole-based drug. Here, we report a simple microwave assisted method for the synthesis of 1,2-disubstituted benzimidazoles. The combination of the molar ratio of *N*-phenyl-*o*-phenylenediamine:benzaldehyde (1:1) using microwave irradiation and only 1% mol of Er(OTf)_3_ provides an efficient and environmental mild access to a diversity of benzimidazoles under solvent-free conditions. The proposed method allows for the obtainment of the desired products in a short time and with very high selectivity.

## 1. Introduction

The use of classic solvents in organic synthesis, and their applications in the pharmaceutical industry, is a strong limitation for environment and human health. In the last years, green chemistry principles influenced the activities of drug industries, introducing the use of new eco-sustainable solvents [1,2,3] and recyclable reagents and reducing in waste production [4,5,6,7,8]. 

In this regard, numerous studies have been performed on the use of environmental solvents [9,10,11,12], bio-sourced ingredient-based solvents [13,14], ionic liquids [15,16,17], deep eutectic solvents [18,19,20,21,22,23], supercritical fluids [24,25], and water [26,27,28,29,30,31,32,33]. Furthermore, solvent free reaction conditions contribute to the sustainability of the entire production system by greatly reducing industrial waste. These reaction methods may be conducted using the reactants alone, or they may involve the use of solid supports (clays, zeolites, silica, alumina, or other matrices). The experimental procedures are easier and have a faster-improving yield, considerably lowering the environmental impact [34,35]. 

The reactions in solvent-free conditions under ultrasonic [36] or microwave [37] irradiation play a very important role in eco-sustainable extraction [38,39] and synthesis [40,41,42,43,44], because they greatly prevent waste, and often only irradiation is useful for activating the organic reaction.

Due to their properties and applications, benzimidazoles are a class of heterocyclic compounds of great interest in the pharmaceutical chemistry area. The benzimidazole ring constitutes the basic structure of important and different pharmaceutical agents [45,46,47,48,49], such as the vitamin B_12_ [50]. For this reason, the synthesis of benzimidazole derivatives had considerable interest in the development of organic synthetic processes that are applicable on an industrial scale with a low environmental impact.

Recent research on the use of eco-sustainable solvents in organic chemistry for the synthesis of benzimidazoles [51,52,53] has had great prominence in the scientific community, as well as the use of Lewis acid catalysis exploitation homogeneous catalysts [54,55,56,57] in mild reaction conditions. At the same time, experimental reactions using solid supports in conventional solvents [58, 59], in green solvents [60,61], or under solvent-free conditions [62,63] performed, as well as the use of heterogeneous catalysts under solvent-free conditions [64] has been particularly important for the eco-sustainable synthesis of benzimidazoles.

However, the synthetic procedure for the synthesis of 1,2-disubstituted benzimidazole derivatives requires the use of MK10 20% wt with a selectivity that is not always high. Therefore, the synthetic method has often involved the use of purification systems to obtain the desired 1,2-substituted benzimidazole derivative [64].

Considering our experience in Lewis acid catalysis and testing the catalytic activity of Er (III) in reactions under microwave irradiation [65,66] in the benzimidazoles [22,67] and benzodiazepine [68,69] derivative synthesis, we report the development of new, ecofriendly and mild method MW-assisted for the synthesis of a variety of substituted benzimidazoles. The synthetic method does not require the use of solvents, but requires the use of only 1% Er(OTf)_3_ as a catalyst for the formation of benzimidazole derivatives.

## 2. Results

In our initial experiment, we chose *N*-phenyl-*o*-phenylenediamine (1 mmol) and benzaldehyde (1 mml) as starting materials in the different green solvent at different temperatures to obtain the respective disubstituted benzimidazole derivative 1a (Table 1). The initial reactions were tested in environmentally friendly solvents different temperatures. We tested the effect of temperature on the model reaction (Table 1, entry 1) using ethyl lactate as the solvent. The reaction mixture was executed by monitoring the reaction by thin layer chromatography (TLC) and gas chromatography/mass spectrometry (GC/MS) analyses. After two hours, we did not observe any trace of the desired product.

The temperature effects showed that by increasing the reaction temperature to 100 °C, yields are higher but insufficient (Table 1, entry 3). 

At room temperature, using water as a solvent, the GC/MS analysis showed the low conversion of the reagents within 2 h, even when increasing the temperature at 60° (Table 1, entries 4 and 5). The GC/MS analysis showed the presence of the 1,2-disubstituted benzimidazole derivative with higher yields (an increase of 59.6%) at 60 °C in only 120 min (entry 6 in Table 1) and, at a higher temperature in the same reaction time (100 °C, 120 min) (Table 1, entry 7), the reaction yield increased considerably (89.7%). When the mixture reaction was subjected to microwave irradiation, we obtained a good yield in only ten minutes at 60 °C (Table 1, entry 8). Interestingly, the result was obtained when the reaction was carried out (Table 1, entry 9). At this point, exploiting the activity of microwave radiation for activating the organic reactions in the solvent free condition, a good conversion of *N*-phenyl-*o*-phenilendiammine in the desiderated product was observed, obtaining the reaction product with an increase in the yield (89.6%) after only 15 min (Table 1, entry 10). The model reaction showed the complete conversion of *N*-phenyl-*o*-phenilendiammine when the same reaction was performed in a solvent free condition at 60 °C in only 5 min, adding only 1% Er(OTf)_3_ at the mixture reaction (Table 1, entry 10). If the mixture reaction was performed using only 0.5% Er(OTf)_3_ we did not observe the complete conversion of amine after 7 min (Table 1, entry 11). Considering our experience in the use of lanthanide triflates and, in particular, of Er (III) and Ce (III) [70], using Ce(OTf)_3_ in the same molar percentage (1% mol), the reaction showed the complete conversion of amine after 7 min. Considering the higher cost of Ce (OTf)_3_ compared to Er (OTf)_3_, we continued with the synthesis of different disubstituted benzimidazoles using only 1% of Er (III) under MW irradiation. The product and catalyst are separated in two phases after the addition of water and the simple extraction of the product with ethyl acetate.

The only reagents used to obtain the respective crude product in faster reaction times are the aldehyde and *N*-phenyl-*o*-phenylenediamine. MW-activation for the benzimidazole formation reduces the reaction times (from 60 min to 5 min) and enhances the yield as well (from 61.4% to 99.9%).

At this point, the experimental procedure was applied to different aldehydes to obtain the related disubstituted benzimidazoles, and quantitative yields superior to 96% were obtained in all cases (Table 2). 

The high-yield reaction was reported using different substituted benzaldehydes, such as *p*-methyl, *p*-methoxy, and *o*-hydroxy benzaldehyde (entries 2, 3, and 4, Table 2). The reactions performed with *p*-chloro, *p*-fluoro *p*-nitro benzaldehyde, aldehydes containing electron withdrawing groups, (entries 5, 6, and 7, Table 2) afford the corresponding disubstituted benzimidazoles (**4a**–**7a**) in good yields (detected by GC/MS) but with longer reaction times (after 15 min). 

As shown in Table 2, this new method maintained high catalytic activity on various substituted benzaldehydes, alkyl aldehydes, and cinnamaldehydes (entries 8, 9, and 10). The performed reactions using *N*-benzyl *o*-phenylenediamines as *N*-alkyl-*o*-phenylenediamines allowed to obtain the desired benzimidazole under the same conditions with the same reaction times and the same yield (Table 2, entries 12, 13, and 14).

The use of the irradiation microwave has made the reaction process even more green than the previous methodologies, for faster reaction times and for the greater selectivity of product formation. In the development of a green synthetic procedure, the isolation of the product is an additionally significant point. In our method, the benzimidazole derivatives could easily be isolated by the simple addition of water and the extraction with ethyl acetate, a green solvent.

Then, we also found it necessary to demonstrate the potential industrial applicability of this eco-friendly procedure. The preliminary reaction to give **1a** was carried out in a large scale (20 mmol of *N*-phenyl-*o*-phenylenediamine and 20 mmol of benzaldehyde). The reaction was completed in 25 min with an excellent yield (93%) after a simple water addition and an extraction with ethyl acetate. 

In conclusion, a fast, cheap, green, and simple procedure has been developed for the synthesis of benzimidazoles. All reactions were performed in short reaction times (5–10 min) and with reaction yields of 86 to 99% (Table 2). The microwave assistance was fundamental to obtain the product in a quantitative yield.

Unlike the method reactions reported in the literature, the procedure described does not require for the use of solvents but only microwave irradiation to perform the complete reaction process. The proposed method reduces the reaction time and energy consumption, making developing the process industrially appropriate.

## 3. Materials and Methods 

### 3.1. General Methods

All reactions were monitored by gas chromatography/mass spectrometry (GC/MS, Shimadzu workstation). It was constituted by a GC 2010 (equipped with a 30 m-QUADREX 007-5MS capillary column, operating in the “split” mode, with 1 mL min-1 flow of He as carrier gas).

^1^H-NMR and ^13^C-NMR spectra were recorded at 300 MHz and at 75 MHz, respectively, using a Bruker WM 300 system. The samples were solubilized in CDCl_3_ using tetramethylsilane (TMS) as a reference (δ 0.00). Chemical shifts are given in parts per million (ppm) and coupling constants (J) are given in hertz. For ^13^C-NMR, the chemical shifts are relative to CDCl_3_ (δ 77.0).

The Synthos 3000 instrument from Anton–Paar, equipped with a 4 × 24MG5 Rotor, was used for the MW-assisted reactions. An external IR sensor monitored the temperature at the base of each reaction vessel. 

### 3.2. General Procedure for the Synthesis of 1-phenyl-2-Aryl(alkyl) Benzimidazoles ***1a***–***11a***

To the *N*-phenil-*o*-phenilendiammine (1 mmol) and Er(OTf)_3_ (1% mol) in a 3 mL glass, aryl o alkyl aldehyde (1 mmol) was added. The mixture reacted for 5 min in a Synthos 3000 microwave instrument, fixed on a temperature value of 60 °C (IR limit). The reaction was monitored by TLC and GC/MS analyses. After the completion of the conversion of *N*-phenil-*o*-phenilendiammine, the Er(OTf)_3_ was separated from the reaction mixture by adding water (to separate the catalyst from the reaction mixture) and extracting the organic product with ethyl acetate (4 × 3 mL). The products were isolated after its organic phases and was dried over Na_2_SO_4_, followed by evaporation under reduced pressure (**1a**–**10a** in 91–99% yields). Spectral data were in accordance with the literature [71]. See Supplementary Materials.

### 3.3. General Procedure for the Synthesis of 1-benzyl-2-Aryl-Benzimidazoles ***1b***–***3b***

To the *N*-benzyl-*o*-phenilendiammine (1 mmol) and Er(OTf)_3_ (1% mmol) in a 3 mL glass, benzaldehyde or *p*-substituted-benzaldehyde (1 mmol) was added. The mixture reaction was reacted in the same reaction conditions that have been previously reported (MW irradiation for 5 min). After the completion of the conversion of *N*-phenil-*o*-phenilendiammine, the Er(OTf)_3_ was separated from the reaction mixture adding water and extracting the organic product with ethyl acetate (4 × 3 mL). The products were isolated after organic phases dried over Na_2_SO_4_, followed by evaporation under reduced pressure. Spectral data were in accordance with the literature [72,73,74]. See Supplementary Materials.

## 4. Conclusions

In summary, the current research shows a rapid, cheap, clean, and environmentally sustainable method of the microwave-assisted synthesis of 1,2-bisubstituted benzimidazoles. The procedure does not require the use of a solvent and has a simple product recovery.

The use of the Lewis catalyst Er(OTf)_3_ (1% mmol) provides a synthetic procedure which considerably reduces reaction times and waste reactions, further promoting the green chemistry principles and industrial applications.

## Figures and Tables

**Table 1:**
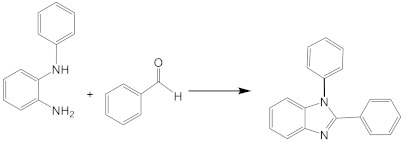
Optimization of the reaction conditions ^a^.

c	Solvent	Temp (°C)	Time (min)	Yield (%) ^b^
1	Ethyl lactate	rt	120	0
2	Ethyl lactate	60	120	3.9
3	Ethyl lactate	100	120	15.3
4	Water	rt	120	10.2
5	Water	60	60	20.9
6	Water	60	120	59.6
7	Water	100	120	89.6
8 ^c^	Water	60	10	71.9
9	-	60	60	61.4
10 ^c^	-	60	5	89.6
**11 ^c,d^**	**-**	**60**	**5**	**99.9**
12 ^c,f^	-	60	7	91.3
12 ^c,g^	-	60	7	99.9

^a^ General reaction conditions: *N*-phenyl-*o*-phenylenediamine (1 mmol) and benzaldehyde (1 mmol) were stirred for 5–120 min at different temperatures in appropriate solvent. ^b^ Percent yield calculated from GC/MS data of the corresponding disubstituted benzimidazole derivative. ^c^ Reaction mixture under MW irradiation. ^d^ Er(OTf)_3_ (1% mol). ^f^ Er(OTf)_3_ (0.5% mol). ^g^ Ce(OTf)_3_ (1% mol).

**Table 2:** Synthesis of 1,2-disubstituted benzimidazoles ^a^.

Entry	Aldehyde	Product	Time (min)	Yield (%) ^b^
1	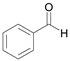	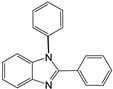 **1a**	5	99.9
2	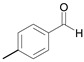	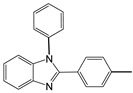 **2a**	5	98.6
3	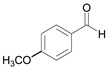	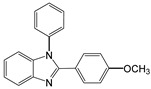 **3a**	7	99.6
4	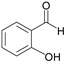	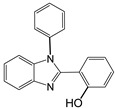 **4a**	10	96.3
5	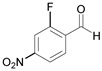	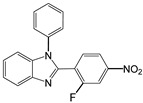 **5a**	15	96
6	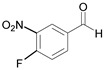	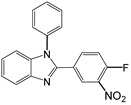 **6a**	15	97
7	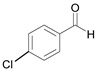	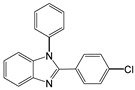 **7a**	10	97
8	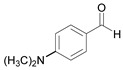	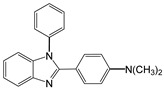 **8a**	15	91.1
9	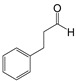	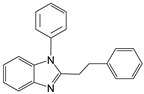 **9a**	5	98.2
10	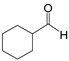	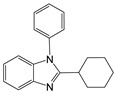 **10a**	5	98.8
11	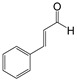	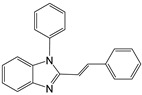 **11a**	12	85.8
12 ^c^	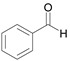	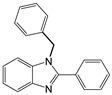 **1b**	5	99.9
13 ^c^	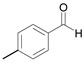	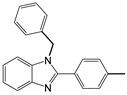 **2b**	5	98.9
14 ^c^	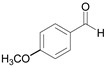	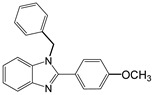 **3b**	5	99.8

^a^ General reaction conditions: The mixture reaction (1 mmol of *N*-phenyl-*o*-phenylenediamine, 1 mmol of aldehyde, and 1% mmol of Er(OTf)_3_) conducted in a Syntos 3000 microwave oven (Anton–Paar) at 60 °C for 5–10 min. The reaction mixture gave the corresponding products **1a**–**8a**. ^b^ Percent yield calculated from GC/MS data. ^c^ The mixture reaction conducted in the same reaction conditions using the *N*-benzyl-*o*-phenylenediamine as *N*-substituted-*o*-phenylenediamine.

## Supplementary Materials

The following supporting information can be downloaded online. Experimental Section, General Procedure for the Synthesis of 1-phenyl-2-Aryl(alkyl) Benzimidazoles 1a-11a, General Procedure for the Synthesis of 1- benzyl-2-Aryl-Benzimidazoles 1b-3b, 1H NMR and 13C NMR of compounds 1a–3a, 6a–8a, ^1^H NMR and ^13^C NMR of compounds 1b–3b.
