# Supplementary material for: The Highly Efficient Synthesis of 1,2-Disubstituted Benzimidazoles Using Microwave Irradiation
Source: Molecules. 2022 Mar 7;27(5):1751. doi: 10.3390/molecules27051751 (PMC8911685; doi:10.3390/molecules27051751)
Supplement: Supplementary file 1 [file molecules-27-01751-s001.zip › molecules-1611895-supplementary.pdf]

The Highly Efficient Synthesis of 1,2-Disubstituted Benzimidazoles Using Microwave Irradiation

Monica Nardi [1,*], Sonia Bonacci [1], Natividad Herrera Cano [2], Manuela Oliverio [1] and Antonio Procopio [1]

[1] Dipartimento di Scienze della Salute, Università Magna Græcia, Viale Europa, Germaneto, 88100 Catanzaro, CZ, Italy; s.bonacci@unicz.it (S.B.); m.oliverio@unicz.it (M.O.); procopio@unicz.it (A.P.)

[2] Departamento Química Orgánica, Facultad de Ciencias Químicas, ICYTAC, CONICET and Universidad Nacional de Córdoba, Ciudad Universitaria, Bv. Juan Filloy s/n, Córdoba 5000, Argentina; nhc@fcq.unc.edu.ar

* Correspondence: monica.nardi@unicz.it; Tel.: +39-0961-3694116

Electronic Supplementary Material

Table of Contents

Experimental Section
General Procedure for the Synthesis of 1,2-Substituted Benzimidazoles 1a-8a.
General Procedure for the Synthesis of 1,2-Substituted Benzimidazoles 1b-3b.
**1-Benzyl-2-phenyl-1*H*-benzimidazole (1b)**
1**-Benzyl-2-(p-tolyl)-1H-benzimidazole (2b)**
**1-Benzyl-2-(4-methoxyphenyl)-1H-benzo[d]imidazole (3b)**

Experimental Section

All reactions were monitored by GC-MS Shimadzu workstation. It is constituted by a GC 2010 (equipped with a 30 m-QUADREX 007-5MS capillary column, operating in the "split" mode, 1 mL min-1 flow of He as carrier gas).

^{1}H-NMR and ^{13}C-NMR spectra were recorded at 300 MHz and at 75 MHz respectively, using a Bruker WM 300 system. The samples solubilized in $CDCl_3$ using tetramethylsilane (TMS) as reference (δ 0.00). Chemical shifts are given in parts per million (ppm) and coupling constants (J) are given in hertz. For ^{13}C-NMR the chemical shifts are relative to $CDCl_3$ (δ 77.0).

Synthos 3000 instrument from Anton Paar, equipped with a 4 × 24MG5 Rotor, used for the MW-assisted reactions. An external IR sensor monitors the temperature at the base of each reaction vessel.

General Procedure for the Synthesis of 1-phenyl-2-Aryl(alkyl) Benzimidazoles 1a-11a.

To the *N*-phenil-*o*-phenilendiammine (1 mmol) and $Er(OTf)_3$ (1% mol) in a 3 mL glass via, the aryl o alkyl aldehyde (1 mmol) was added. . The mixture was reacted for 5 min in a Synthos 3000 microwave instrument, fixed on a temperature value of 60 °C (IR limit). The reaction was monitored by TLC and GC/MS analysis. After completion conversion of *N*-phenil-*o*-phenilendiammine, the $Er(OTf)_3$ was separated from the reaction mixture adding water (to separate the catalyst from the reaction mixture)and extracting the organic product with ethyl acetate (4x3 mL). The products were isolated after organic phases dried over Na_2SO_4, followed by evaporation under reduced pressure (1a–10a in 91–99% yields). Spectral data were in accordance with the literature [71]

General Procedure for the Synthesis of 1- benzyl-2-Aryl-Benzimidazoles 1b-3b.

To the *N*- benzyl-*o*-phenilendiammine (1 mmol) and $Er(OTf)_3$ (1% mmol) in a 3 mL glass via, the benzaldehyde or *p*-sostituited-benzaldehyde (1 mmol) was added. The mixture reaction was reacted in the same reaction conditions previously reported (MW irradiation for 5 min). After completion conversion of *N*-phenil-*o*-phenilendiammine, the $Er(OTf)_3$ was separated from the reaction mixture adding water and extracting the organic product with ethyl acetate (4x3 mL). The products were isolated after organic phases dried over Na_2SO_4, followed by evaporation under reduced pressure. Spectral data were in accordance with the literature [72-74].

1-Benzyl-2-phenyl-1*H*-benzimidazole (1b): White solid; m.p. 132–134 °C [72], ^{1}H NMR (300 MHz, $CDCl_3$, δ ppm (J, Hz): 7.87 (d, J=7.8 Hz, 1 H), 7.71 (d, J=7.8 Hz, 2 H), 7.50–7.44 (m, 3 H), 7.34–7.20 (m, 6 H), 7.10 (d, J=6.7 Hz, 2 H), 5.48 (s, 2 H); ^{13}C NMR (75 MHz, $CDCl_3$) δ ppm: 154.2, 143.1, 136.2, 136.0, 130.0, 129.7, 129.1, 129.0, 128.5, 127.6, 125.8, 122.8, 122.5, 119.8, 110.1, 48.2. Anal. Calcd for C20H16N2: C, 84.50; H, 5.63; N, 9.85 Found: C, 84.51; H, 5.69; N, 9.80

1**-Benzyl-2-(p-tolyl)-1H-benzimidazole (2b):** White solid. Mp: 129-131° [78] C. ^{1}H-NMR (300 MHz, $CDCl_3$): δ = 7.87 (d, J = 8.0 Hz, 1H), 7.59 (d, J = 8.0 Hz, 2H), 7.31-7.27 (m, 4H), 7.24 (d, J = 8.0 Hz, 2H), 7.21-7.19 (m, 2H), 7.01 (d, J = 6.8 Hz, 2H), 5.43 (s, 2H), 2.39 (s, 3H); 13C NMR (75 MHz, $CDCl_3$): δ = 154.34, 143.15, 140.10, 136.51, 136.09, 129.48, 129.17, 129.06, 127.75, 127.13, 125.98, 122.91, 122.63, 119.88, 110.49, 48.39, 21.45. HRMS calcd for C21H18N2 [(M+H)+]: 299.1543; found, 299.1550.

1-Benzyl-2-(4-methoxyphenyl)-1H-benzmidazole (3b). White solid . Mp: 133-135 °C [79]. ^{1}H-NMR (300 MHz, $CDCl_3$): δ = 7.86 (d, J = 8.0 Hz, 1H), 7.63 (d, J = 8.4 Hz, 2H), 7.33-7.24 (m, 2H), 7.21 (dd, J = 14.8 Hz, 8.0 Hz, 2H), 7.09 (d, J = 6.8 Hz, 2H), 6.96-6.93 (m, 2H), 5.41 (s, 2H), 3.81 (s, 3H); ^{13}C NMR (75 MHz, CDCl3): δ = 160.95, 154.18, 143.22, 136.55, 136.16, 130.70, 129.08, 127.75, 125.96, 122.79, 122.59, 122.41, 119.76, 114.23,110.40, 55.37, 48.38. HRMS calcd for C21H18N2O [(M+H)+]: 315.1492; found, 315.1496.

1-Benzyl-2-phenyl-1*H*-benzimidazole (1b):

^{1}H-NMR

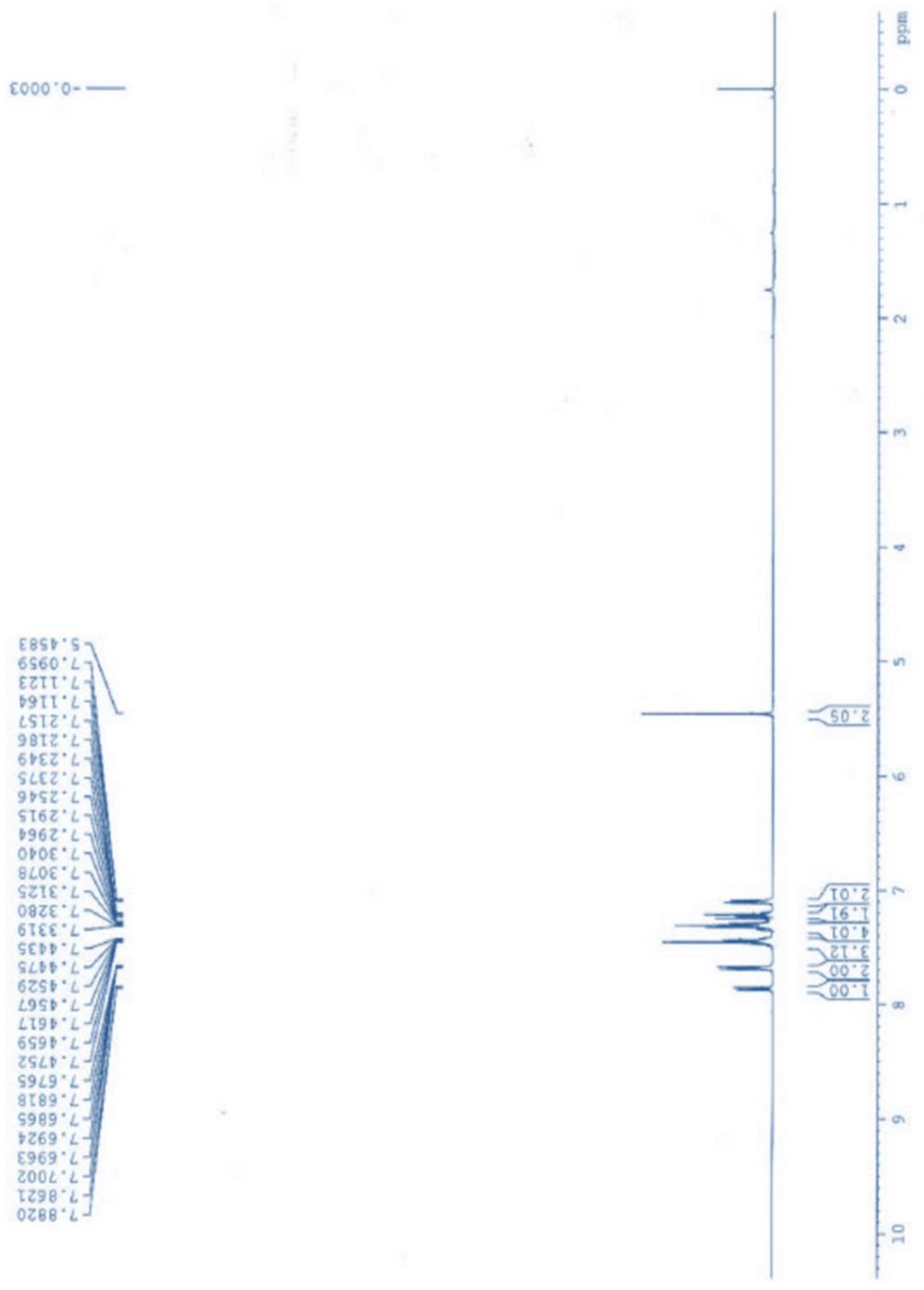
-0.0003
5.4583
7.0959
7.1123
7.1164
7.2157
7.2186
7.2349
7.2375
7.2546
7.2915
7.2964
7.3040
7.3078
7.3125
7.3280
7.3319
7.4435
7.4475
7.4529
7.4567
7.4617
7.4659
7.4752
7.6765
7.6818
7.6865
7.6924
7.6963
7.7002
7.8621
7.8820
2.05
2.01
1.91
4.01
3.12
2.00
1.00
ppm
0
1
2
3
4
5
6
7
8
9
10

^{13}C-NMR

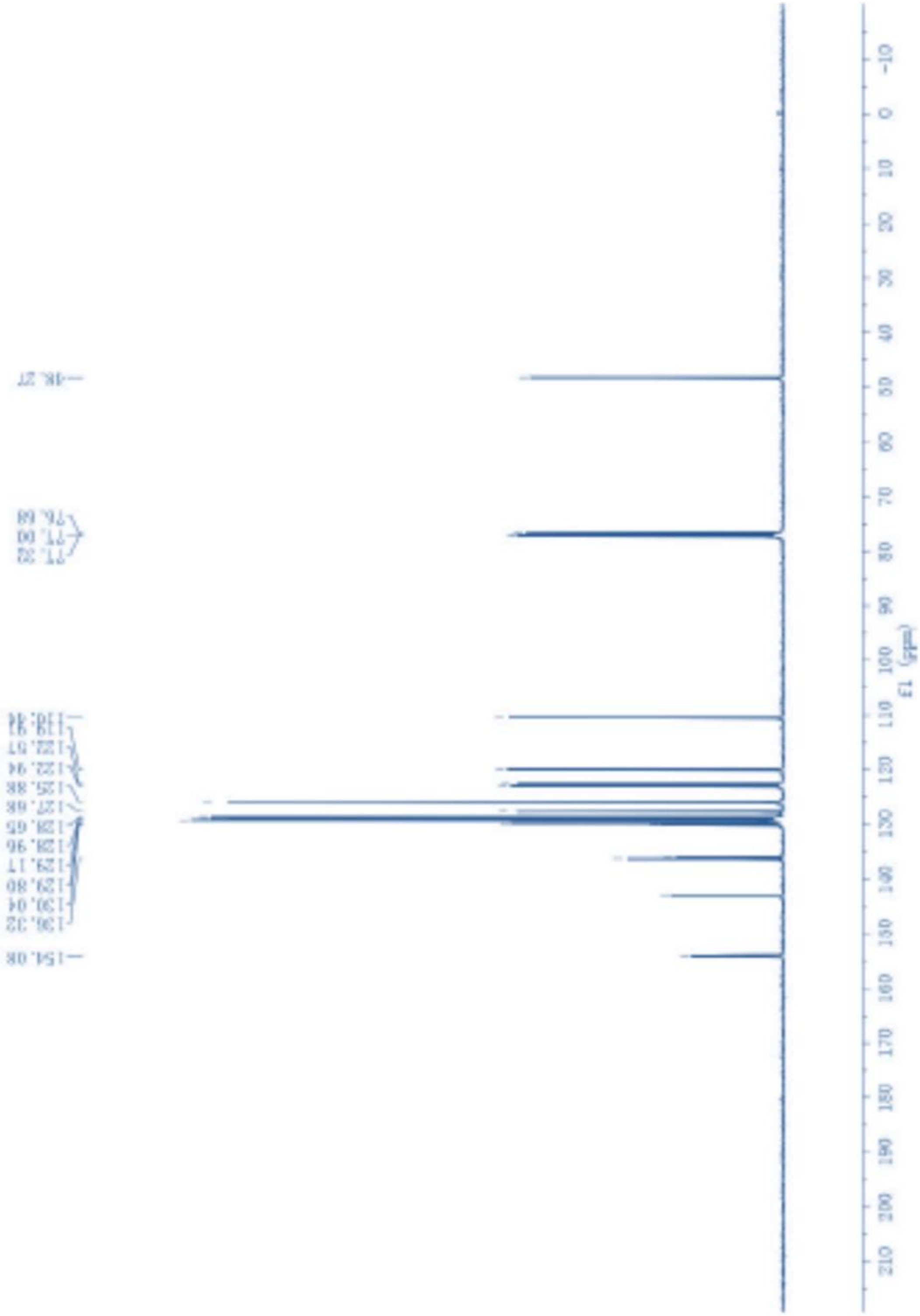

1-Benzyl-2-(p-tolyl)-1H-benzimidazole (2b)

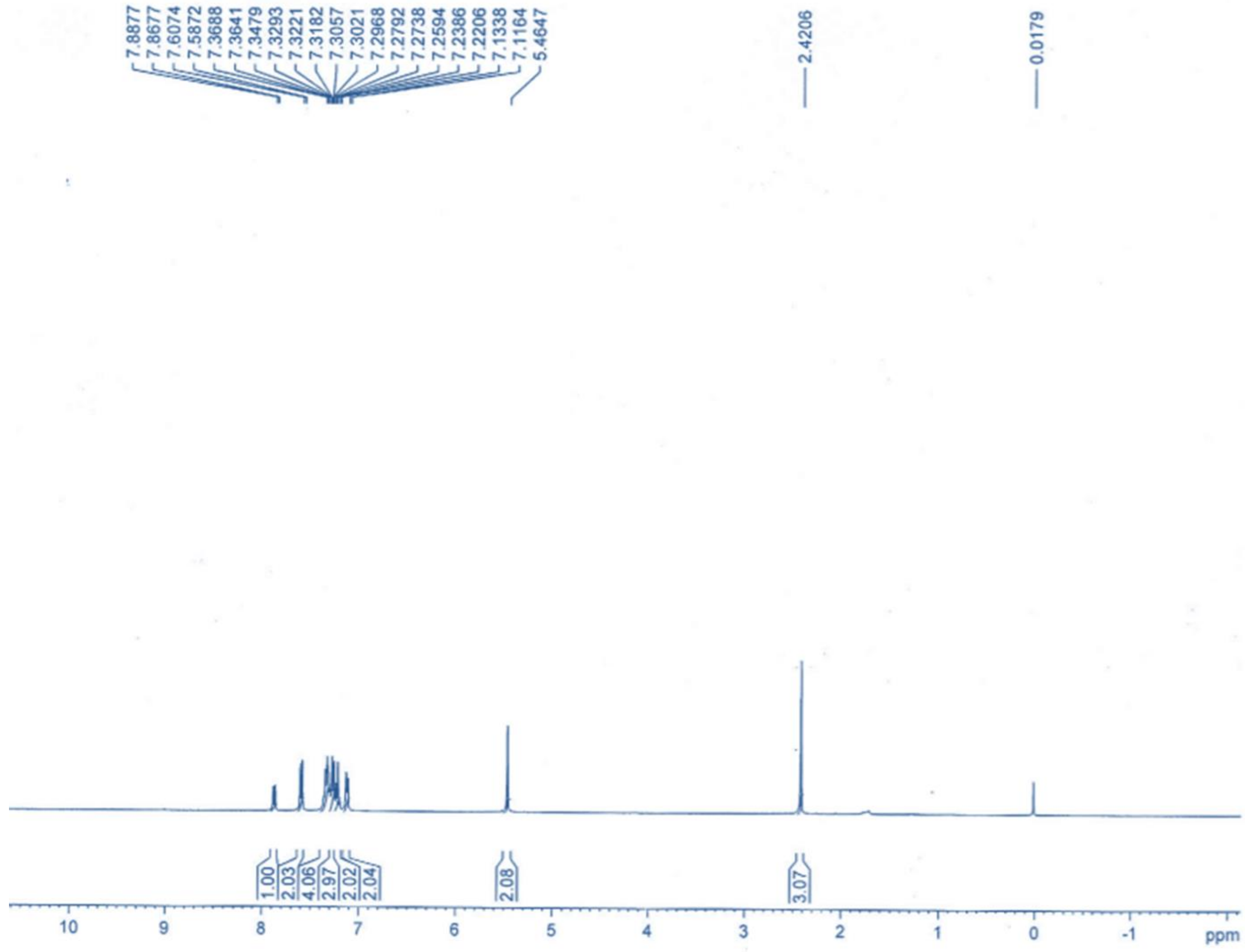
7.8877
7.8677
7.6074
7.5872
7.3688
7.3641
7.3479
7.3293
7.3221
7.3182
7.3057
7.3021
7.2968
7.2792
7.2738
7.2594
7.2386
7.2206
7.1338
7.1164
5.4647
2.4206
0.0179
1.00
2.03
4.06
2.97
2.02
2.04
2.08
3.07
10
9
8
7
6
5
4
3
2
1
0
-1
ppm

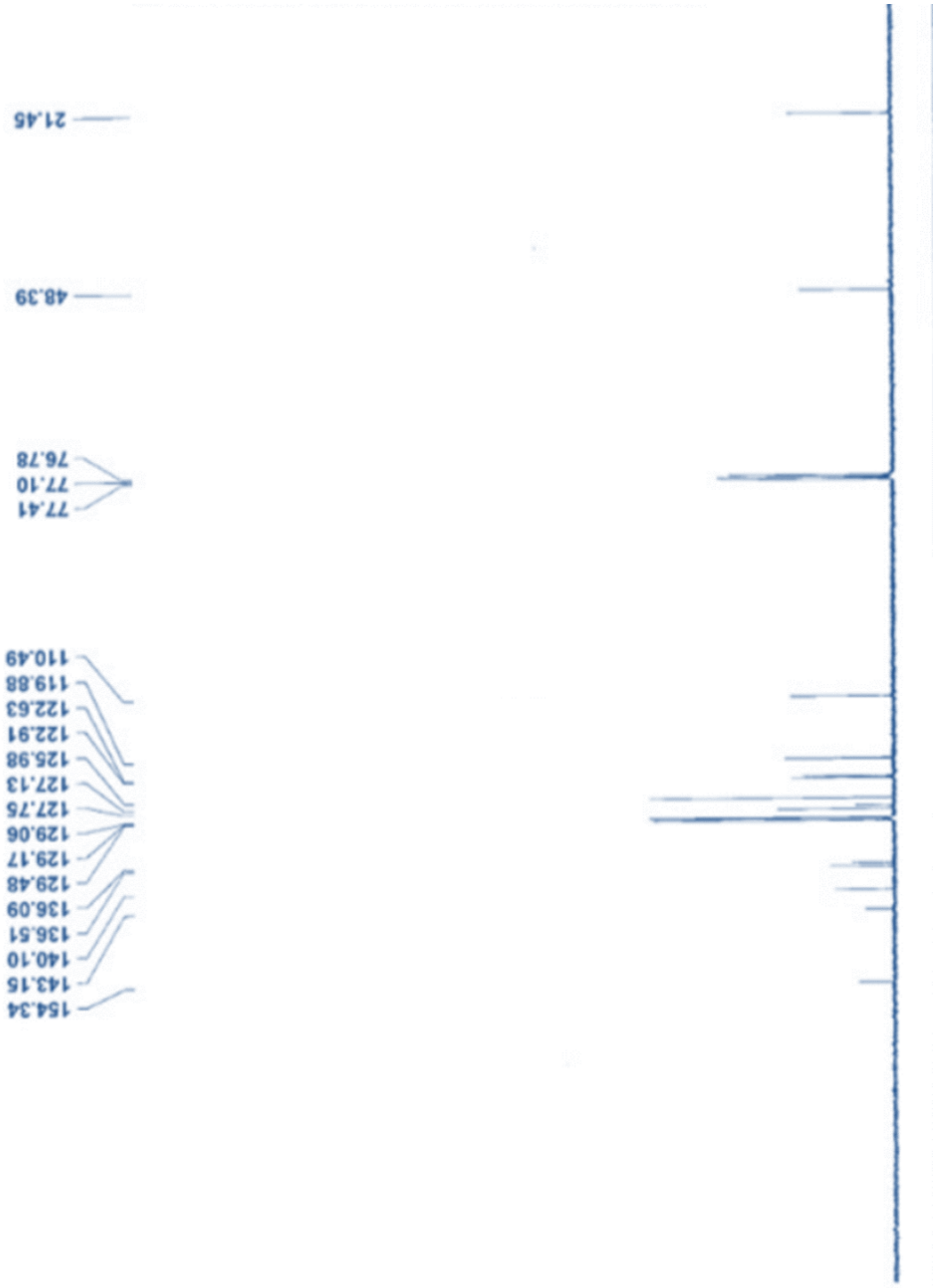

1-Benzyl-2-(4-methoxyphenyl)-1H-benzmidazole (3b).

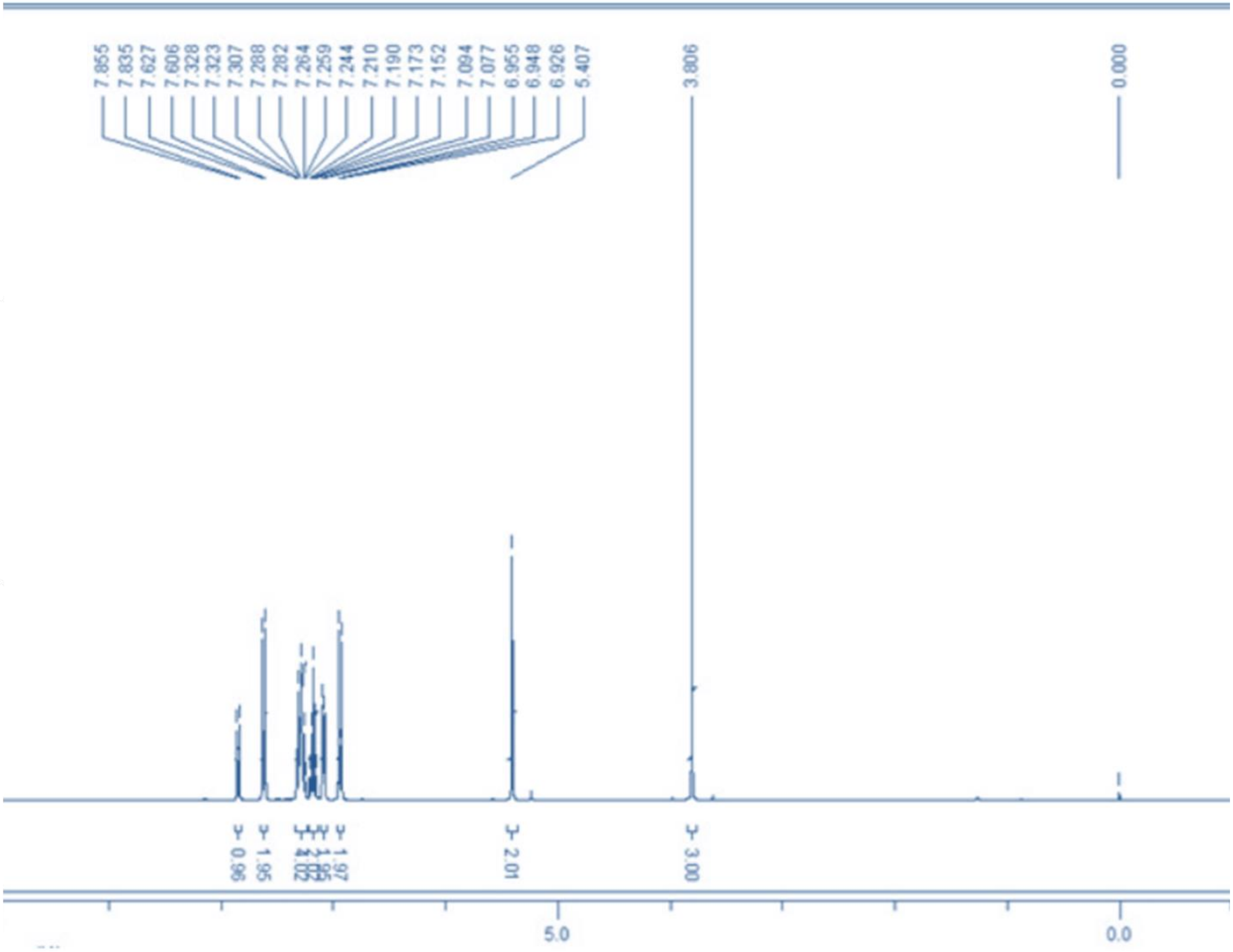
7.855
7.835
7.627
7.606
7.328
7.323
7.307
7.288
7.282
7.264
7.259
7.244
7.210
7.190
7.173
7.152
7.094
7.077
6.955
6.948
6.926
5.407
3.806
0.000
0.96
1.95
4.02
1.97
2.01
3.00
5.0
0.0

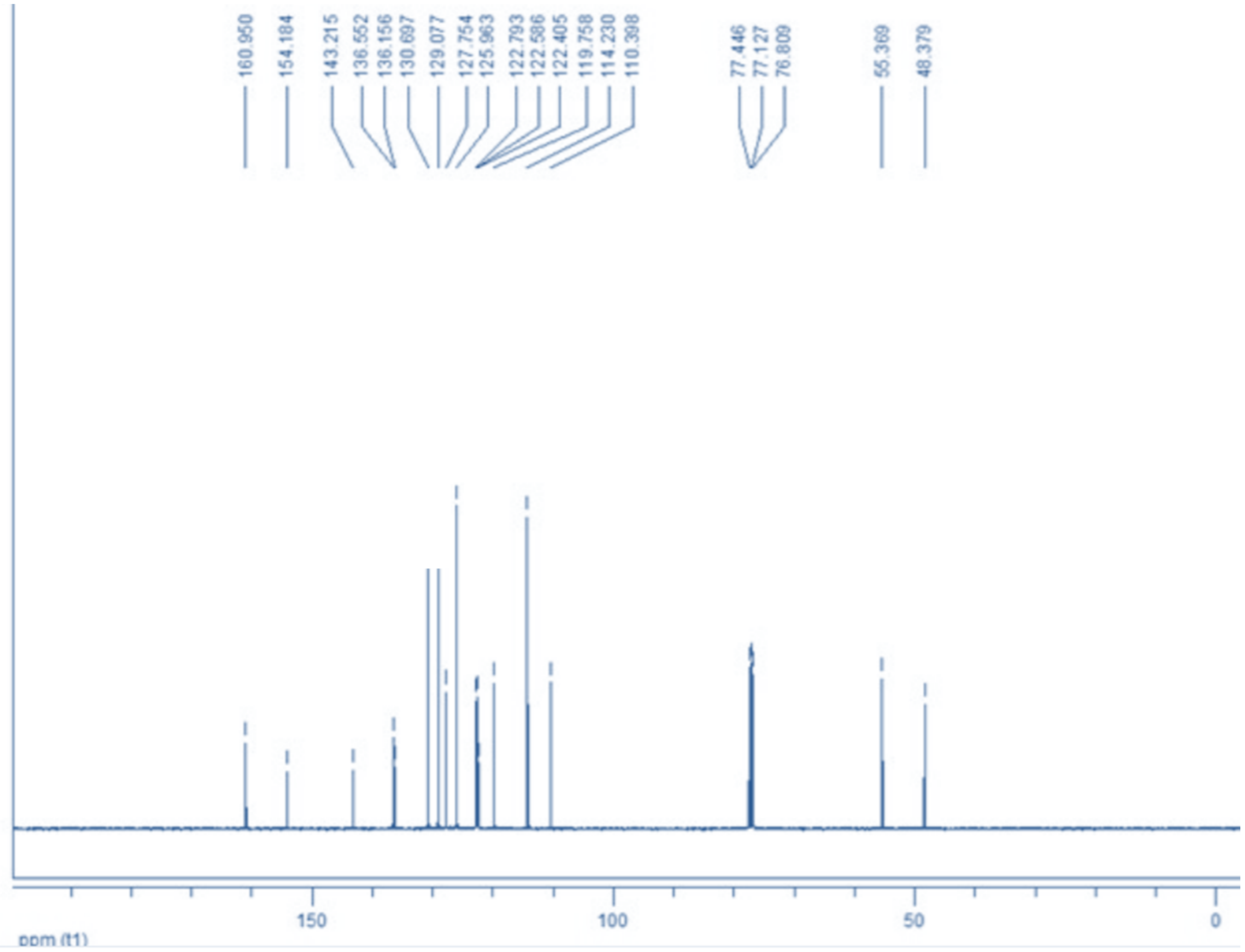
160.950
154.184
143.215
136.552
136.156
130.697
129.077
127.754
125.963
122.793
122.586
122.405
119.758
114.230
110.398
77.446
77.127
76.809
55.369
48.379
150
100
50
0
ppm (t1)
